# Efficacy of depression treatments for immigrant patients: results from a systematic review

**DOI:** 10.1186/1471-244X-14-176

**Published:** 2014-06-16

**Authors:** Josefine Antoniades, Danielle Mazza, Bianca Brijnath

**Affiliations:** 1 Department of General Practice, Monash University, Building 1, 270 Ferntree Gully Rd, Notting Hill, VIC 3168, Australia

**Keywords:** Migrant, Depression, Intervention, Review

## Abstract

**Background:**

The unprecedented rates of global migration present unique challenges to mental health services in migrant receiving countries to provide efficacious and culturally salient treatment for mental health conditions including depression. This review aimed to identify and evaluate the effectiveness of depression interventions specifically directed towards first-generation immigrant populations.

**Methods:**

We conducted a systematic review of original research published between 2000 and 2013 that investigated depression interventions in first generation immigrants.

**Results:**

Fifteen studies were included; the majority focused on Latino immigrants living in the United States (US). Twelve studies investigated the use of psychotherapies; the remainder examined collaborative care models and physical exercise-based interventions. Cognitive Behavioral Therapy and Behavioral Activation tended to improve depressive symptoms, especially when culturally adapted to suit clients while Problem Solving Therapy improved depressive symptomology with and without adaptations. Collaborative care and exercise did not significantly improve depressive symptoms.

**Conclusion:**

Depression may be effectively treated by means of psychotherapies, especially when treatments are culturally adapted. However the reviewed studies were limited due to methodological weaknesses and were predominantly undertaken in the US with Latino patients. To improve generalizability, future research should be undertaken in non-US settings, amongst diverse ethnic groups and utilize larger sample sizes in either randomized clinical trials or observational cohort studies.

## Background

Depression is the leading cause of global disability affecting nearly 350 million people worldwide. It disproportionately affects women and at its most severe can result in suicide [1]. Though treatable in primary and acute care settings through a range of psychosocial therapies and medication, less than half those suffering from depression are diagnosed and receive treatment [1,2].

Migrants are an example of a population group where there is under diagnosis and low treatment of depression [3]. Reasons for this include difficulties in assessment and social stigma associated with depression, variance in depression symptomology, professional nosologies, patterns of help-seeking and self-management see for example [3-7]. These factors, singularly and collectively, can delay timely diagnosis and treatment for migrants, which is problematic because migrants are a rapidly growing population cohort in nearly all industrialized countries [8]. The current trend of increased global migration as well as the projected rise in mental illness, in particular depression, necessitate forward-planning and strategic service delivery in order to achieve equitable access to mental health services for all.

The challenge currently being faced by health services in immigrant-receiving countries is in planning and delivering appropriate, evidence based, and where possible, culturally salient mental health care to increasingly diverse populations [8,9]. Unfortunately, the evidence-base that policy makers and service providers may draw on to guide the development of more culturally salient depression treatment interventions is limited in two ways. First, research investigating depression treatment in immigrant populations is sparse and treatment recommendations are largely inferred on the basis of studies done on Caucasian populations [10]. Second, while there is evidence to suggest that culturally framed interventions are effective in treating mental disorders in culturally diverse patient groups [11], it is not clear which specific models and interventions are more (or less) effective in treating depression in immigrant populations [12]. Therefore, this review will systematically identify studies that investigate depression treatments in immigrant populations and evaluate the effectiveness of these treatment strategies/models.

## Method

### Search strategy

The literature search covered six databases: MEDLINE, PsychINFO, EMBASE, Cochrane Central Register of Controlled Trials, CINAHL and Web of Knowledge. Keywords to identify studies included DEPRESSION (depress*, depressed, depression), IMMIGRANT (immigra*, refuge*, asylum seek*, ethnic minorit*, latin*, migrant*) and TREATMENT (treatment, interven*, therap*). The search was limited to English language peer–reviewed articles published between 2000 and 2013 as we aimed to review the most current evidence.

All articles returned by the database search were screened to assess relevance to the aims and a provisional reference list was compiled. Following the database search, a grey literature search was conducted using Google Scholar and identified studies’ reference lists were also reviewed to identify additional studies of interest. Any relevant references were added to the provisional reference list.

### Study inclusion and exclusion criteria

Studies were included if they met the following criteria: (1) they reported original research of either a quantitative, qualitative or mixed methods design, (2) they described a treatment - pharmacological, psychological or otherwise - designed to reduce depressive symptoms, (3) the treatment specifically targeted first-generation immigrant populations, (4) the studies’ participants were 18 years or above and (5) the studies’ participants were diagnosed with depression or met the criteria for depression on a validated depression screening tool administered for the purpose of the study.

Studies were excluded if participants were non-immigrants or second or subsequent generation migrants, under the age of 18, or if comorbidities were reported including other mental illnesses or physical conditions. We excluded second and subsequent generations of migrants because the literature shows that these cohorts are generally more acculturated than first generation migrants and that their health status is very similar to the local population [13-15]. Studies that included participants under the age of 18 were excluded. This was done to avoid the additional and unique complexities associated with child and adolescent mental health. Similarly we excluded studies that reported on comorbidities (e.g. cancer and depression) in order to disentangle conclusions about depression treatment from interventions targeting other comorbidities, as these may have added confounding variability in the data presented.

### Screening and assessment

**Figure 1:**
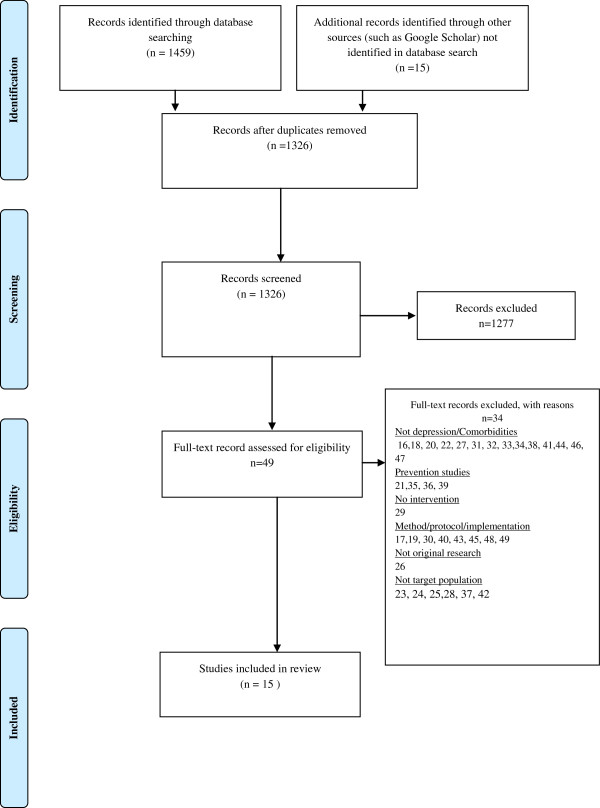
PRISMA flowchart of literature search process.

1326 potential records were identified (Figure 1). After removing duplicates, reviewing titles and abstracts, 49 records were retained for further assessment. These titles, keywords, abstracts and full-text were then reviewed to assess suitability for inclusion in accordance to the inclusion and exclusion criteria. Based on initial assessment 34 of these 49 records were subsequently excluded [16-49] (see Figure 1 for reasons).

Fifteen records were included in the final analysis [50-64]. The abstracts and full texts of these 15 studies were assessed by two researchers independently. To ensure methodological rigor in the review process, all quasi-experimental and experimental studies were appraised for quality in accordance with the United Kingdom’s National Institute for Health and Care Excellence (NICE) guidelines [65]. Non-experimental studies were assessed using critical appraisal forms adopted from Crombie [66] and used by the Oxford Centre for Evidence Based Medicine, BMJ and Dutch Cochrane Centre [67]. Any disagreement between assessments after full text review was resolved through consensus.

## Results

### Study characteristics

Of the 15 studies, nine were quantitative [51-54,56,57,60,63,64], five employed mixed methods [50,55,58,59,62] and one case study presented qualitative data [61]. Nine studies reported on culturally sensitive/culturally adapted psychological treatments (Table 1). Substantial variation in sample sizes as well as intervention duration across the studies was observed (Table 1). Most studies offered the intervention in the preferred language of the target group or made use of interpretative services (Table 1).

**Table 1:** Intervention summary and results

**Study**	**Intervention**	**Language**	**Control**	**Design**	**N**	**Data collection**	Primary outcome measure*	**Effect on depressive symptoms**	**Sig.**
Yeung *et al.* (2010) [64]	Culturally-sensitive, collaborative treatment (8 sessions/24 weeks)	English/Chinese	UC^a^	Descriptive uncontrolled design	100	6 months, follow up: 1.5, 3.5 and 6 months	HAM-D CGI-S CGI-I	Decrease in depressive symptoms in Control and Intervention. No significant difference between groups	No
Choi *et al.* (2012) [53]	Culturally-adapted, internet CBT (8 weeks)	English/Chinese	WL^b^	RCT	63	Pre, post and 3 month (intervention only)	CBDI CB-PHQ9	Large effect size on BDI (*d =1.41; d =* .93 within and between groups) and medium to large effect size on CB-PHQ-9 (*d = .90; d =* .50 within and between groups)	Yes
Cho *et al.* (2012) [52]	Logo-autobiography (6 sessions/6 weeks)	Korean	UC with and without medicine	Non-randomised experimental research study	40	Pre, post and 4 week follow up (intervention only)	CES-D (Korean)	Depression scores significantly decreased in I relative to C at post-test (*p* = .013) and 4 weeks (*p* = .001). Effect size .50. No significant difference between Imed and Cmed	Partly
Tang *et al.* (2005) [61]	CBT (16 sessions/5 months)	Cantonese	None	Case study	1	Not described	GDS	Depression scores on GDS decreased by 8 points by conclusion of study	NA
Yeung *et al.* (2012) [63]	Tai Chi (2×1 hr/12 weeks)	Chinese	WL	Pilot RCT	39	Baseline, 6 12 weeks	HAM-D CGI-S CGI-I	Non-significant positive trend towards remission of depression	No
Dwight-Johnson *et al.* (2011) [54]	Culturally-tailored, telephone based CBT intervention (8 sessions)	Spanish	Enhanced UC	Randomised pilot study	101	Baseline, 6 weeks, 3 months, 6 months	SCL PHQ-9	Non-significant positive trend towards remission of depression in intervention group	No
Piedra *et al.* (2012) [58]	Group CBT “*Vida alegra”* (10 sessions/10 weeks)	Spanish	None	Pre/post/follow-up study	19	Baseline, Post test, 3 months	CES-D	Significant reduction in CES-D, effect size = .67	Yes
Interian *et al.* (2008) [56]	Culturally-adapted, CBT (12 sessions)	Spanish	None	Pre/post/follow-up study	15	Baseline, Post test, 6 months	BDI-S PHQ-15	Significant reduction in BDI-S scores at post (*p* = .0005) and 6 months (*p* = .0005). PHQ-15 significant at post assessment (*p* = .004), and at 6 months (*p* = .01)	Yes
Kanter *et al.* (2010) [57]	Culturally-adapted, Behavioral Activation (12 sessions)	Spanish/English	UC	Pre/post study design	10	Pre and post (following 12 sessions or 20 weeks which ever came first)	BDI-II HRSD	Significant improvements observed on BDI-II, large effect size *d* = 1.67, HRSD, effect size *d* = 1.57	Yes
Schmaling *et al.* (2008) [60]	Problem Solving Therapy for Primary Care (8 sessions)	Spanish/English	Participants refusing treatment, Non-completers	Pre/post repeated measures study	117	baseline and ~ 4 months	HSCL-20	Significant improvement following 4+ sessions compared to 3 or less sessions, p < .05. ≥4 sessions decrease of m *=* .86 point. ≤ 3 sessions decrease of m = .4 points	Yes
Chu *et al.* (2012) [50]	Culturally-adapted, Problem Solving Therapy (12 sessions)	English	None	Pilot case study	1	Pre and post intervention	PHQ-9 Mood	PHQ-9 score decreased from 12 to 3 Mood improved	NA
Beeber *et al.* (2012) [51]	Culturally-sensitive, home-based IPT (11 in-home sessions with nurse/interpreter, 5 short sessions with interpreter only)	Spanish	Enhanced UC	RCT	80	Baseline (T1), 14 (T2), 22 (T3) (termination) weeks and 4 weeks post termination (T4)	CES-D	Significant improvement in CES-D scores : CES-D within group changes: T1 vs T2, *p* = .021 T1 vs T3, *p* = .005 T1vs T4, *p* = .021	Yes
Gelman *et al.* (2005) [55]	Culturally-adapted, group CBT (12× weekly sessions)	Spanish	None	Pilot pre-post repeated measures study	5	pre and post intervention	BDI-S	BDI scores significantly reduced (*p* = .01)	Yes
Uebelacker *et al.* (2011) [62]	Telephone depression care management (D-HELP) (8 calls/12 weeks)	Spanish	UC	Pilot RCT	38	pre, 6 and 12 weeks post intervention	QUIDS CES-D	Non-significant positive trend towards remission of depression	No
Renner *et al.* (2011) [59]	CBT and Self Help group (SH) intervention (15 session/4 months)	CBT: German with interpreter support SH: Turkish	WL	RCT	38	Pre, termination, 4 weeks, 6 month follow-up	CES-D, BSI PHQ-Turkish	SHG ineffective, CBT decreased depressive symptoms on BSI only and results deteriorated over time	No

*Abbreviations: *Ham-D/HRSD* Hamilton Rating Scale for depression; *CGI-S* Clinical Global Impression Severity Scale; *CGI-I* Clinical Global Impression Improvement Scale; *CBDI* Beck Depression Inventory-I Chinese; *BDI-S* Beck Depression Inventory-Spanish; *BDI-II* Beck Depression Inventory-II; *CB-PHQ9* Chinese Bilingual version of the Patient Health Questionnaire; *PHQ* Patient Health Questionnaire; *CES-D* Center for Epidemiologic Studies Depression Scale; *GDS* Geriatric Depression Scale; *SCL/HSCL-20* Hopkins Symptom Checklist; *QUIDS* Quick Inventory of Depressive Symptoms; *BSI* Brief Symptom Inventory.

^a^UC: Usual Care.

^b^WL: Waitlist.

### Characteristics of the studies’ participants

The major ethnic groups represented in the studies were Latino immigrants living in the United States (US) (53.3%) [51,54-58,60,62] followed by Chinese-American immigrants (33.3%) [50,61,63,64]. The remaining studies focused on Chinese-Australians (7.1%) [53], Korean-Americans (6.7%) [52] and Turkish immigrants living in Austria (6.7%) [59].

There was a underrepresentation of men in the included studies; five of 15 studies specifically recruited only women [51,52,55,58,59], one study reported having no male participants [57] and two case studies did not describe gender as an inclusion criteria but only included female participants [50,61]. Women, on average, represented 81.2% of the study cohorts, though none of the remaining studies sought to exclude males.

No study specified the nature and reasons for migration of the participants and so we were unable to determine whether migration occurred voluntarily or was forced.

### Quality of studies

The reviewed studies were diverse in methodological approaches. In some studies randomization procedures were employed (Table 2) but these procedures were not consistently reported. Although it is generally good practice to have blinded participants and where possible blinded researchers/care providers and assessors, this would not have been feasible in the studies due to the nature of the intervention and the research design used [68]. There were four studies where the research team did ensure that assessors were blinded [54,62-64].

**Table 2:** Randomized study quality indicators

**Author (year)**	**Randomization**	**Allocation masking**	**Attrition**	**Missing data handling**	**Limitations**
Dwight-Johnson *et al.* (2011) [54]	Yes: Stratified permuted-block randomization	Participants: No	Intervention: 16%	Intent-to-treat analysis (ITT) employed	No power calculation
					ITT can increase chance of false positive
		Researchers: No	Control: 30%		
		Outcome Assessor: Yes			
Ueberlacker *et al.* (2011) [62]	Yes: method not described	Participants: No	Intervention: 26%	Not described in detail, but it appears that missing data points have been excluded.	Small sample, risk of attrition bias
		Researchers: No	Control: 42%		
					No power calculation
		Outcome Assessor: Yes			
Yeung *et al.* (2010) [64]	Yes: computer-generated table	Participants: No	Not reported	Not reported	No power calculation
		Researchers: No			
		Outcome Assessor: Yes			
Yeung *et al.* (2012) [63]	Yes: randomized using computer-generated numbers	Participants: No	Intervention: 27%	Used data from week 6 if no data available at week 12. If neither data point available participant was excluded from analysis	Power calculation suggest much larger sample is required
		Researchers: No	Control: 15%		
		Outcome Assessor: Yes			
Choi *et al.* (2012) [53]	Yes: randomization process by independent person	Participants: No	Intervention: 34%	Baseline carried forward	The missing data approach may introduce false positives. No power calculation Small sample
		Researchers: No	Control: 10%		
		Outcome Assessor: No			
Beeber *et al.* (2010) [51]	Yes: block randomization	Participants: No	Intervention: 13%	Power calculation completed and extra participants included to compensate for possible attrition	Small sample
		Researchers: No Outcome Assessor: No	Control: 10%		
Renner *et al.* (2011) [59]	Yes: method not described	Participants: Not reported	Intervention CBT: 52%	Non-completers excluded	Small sample high risk of attrition bias
		Researchers: Not reported	Intervention SHG: 28%		
					Potential risk of selection bias
		Outcome Assessor: Not reported	Control: 45%		
					No power calculation

Depression outcome measures were generally well described, though in a small number of studies details relating to validity and reliability of the outcome measures were not reported [54,55,57,61]. All but one non-experimental study [61] adequately described the interventions being examined as well as control conditions.

The majority of studies reported high attrition rates and missing data handling was generally not well described (Table 2). The most pressing concern regarding missing data handling is the impact that this missing information may have on the direction of the results, hence limiting the generalizability and replicability of the study [69].

Generally, mixed method studies were found to address clearly formulated research questions. It was difficult to ascertain the representativeness of the setting and samples in relation to target populations as most studies recruited participants through various health and community organizations and hence only individuals already engaged with health or community services were included [50,55,57,58,60].

Although all the studies provided valuable information about depression treatment in immigrant populations, there were methodological flaws that in many cases were identified by the authors themselves. A particular difficulty resonating across experimental and non-experimental studies was the difficulty in recruitment and retention of participants from immigration populations, which in turn resulted in small samples and high attrition.

### Interventions and effectiveness

Twelve studies examined the use of psychotherapies either alone, compared to another psychotherapy or waitlist/care as usual conditions. Two studies evaluated the use of collaborative care models and one study investigated the efficacy of a Tai Chi program on depressive symptoms (see Table 3 for qualitative key themes and Table 1 for overview of intervention efficacy).

**Table 3:** Key qualitative themes

**Benefits of therapy**	**Treatment improvements/preferences**
Group therapies provided participants with a sense of community, support and trust [55,59]	Addition of face to face interactions in phone based paradigm [62]
Empowerment through problem solving, coping and interpersonal strategies [55,59,61]	Cultural beliefs about psychotherapy varied in terms of preferences for clinician [59]
Positive experience [59,62]	
Appreciation of personal attention and connection with therapist [55,62]	

### Psychotherapies

#### ***Cognitive Behavioral Therapy***

Cognitive Behavioral Therapy (CBT) was the most commonly investigated psychotherapy; seven studies evaluated the use of CBT in some form. At the core of CBT is cognitive restructuring, interpersonal skills training and engagement in pleasant activities [70]. Currently, CBT is one of the most extensively researched psychotherapies and is widely used in psychiatry as an acute intervention as well as to prevent relapse for depression [71-73]. Although CBT can be delivered by a therapist to an individual or a group, more recently internet and/or phone delivered CBT interventions have become increasingly popular [74].

Studies evaluating CBT varied widely in the method of intervention delivery. Dwight-Johnson *et al.*[54] and Choi *et al.*[53] assessed the efficacy of culturally adapted CBT delivered by phone and internet respectively in randomized trials in which bilingual therapists and translated materials were utilized. Choi *et al.*[53] observed significant reductions in depressive symptoms in Chinese patients on both the Chinese Beck Depression Inventory (CBDI) and Patient Health Questionnaire-9 (CB-PHQ-9) depression items with only marginal drop-out rates reported. The reported with-in and between-group effect sizes (Cohen’s *d*) were 1.41 and *.*93 on the CBDI and .90 and .50 on the CB-PHQ-9 respectively. Conversely, Dwight-Johnson *et al.*[54] only reported a positive trend towards improvement on the Hopkins Symptoms Checklist (SCL) and on the PHQ in Latino participants; however their study was limited because only 44% of participants completed six or more sessions of the eight session intervention (a limitation that they acknowledge).

Two studies investigated therapist-delivered, culturally adapted CBT delivered in participants’ first language [56,61]. Interian *et al.*[56] employed a 12-week culturally adapted CBT intervention that was found to be effective in decreasing depressive symptoms in a small sample (*n =* 15) of Hispanics suffering major depression, immediately post treatment (effect size 2.71) and at the 6 month follow-up point (effect size 2.53). Tang *et al.*[61] reported on the use of CBT to treat depressive symptoms in an elderly Chinese caregiver in a single case study, in which 16 sessions were conducted in Cantonese over five months, which resulted in a decrease in depression scores on the Geriatric Depression Scale (GDS) and greater patient satisfaction with interpersonal relationships.

A group CBT paradigm based on a treatment manual developed by Muñoz *et al.*[70] specifically for Hispanics was examined by Gelman *et al.*[55] and Piedra *et al.*[58] in pre-post and pre/post/follow-up studies respectively. In both studies, therapists were fluent in English and Spanish. Piedra *et al.*[58] reported significant improvement at post-test with effect size of *r*=.67. This concords with findings of Gelman *et al.*[55] who reported an average decrease in Beck Depression Inventory-Spanish (BDI) scores of 12 points (*p* = .01). Qualitative results from both studies suggested great patient satisfaction with the treatment (Table 3).

In a randomized controlled study, Renner *et al.*[59] compared an interpreter-assisted CBT group (intervention) and a self-help group (intervention) facilitated by female, Turkish native speakers to a waitlisted group of Turkish immigrant women (control). The interventions proved ineffective in addressing depressive symptomology. However, this study did not employ a CBT protocol adapted to the target population and therapy sessions were conducted by an Austrian therapist assisted by an interpreter. Qualitative results indicated that participants across both interventions experienced increased mutual trust within the group environment, learned problem solving strategies and felt more emotionally resilient. However, they also indicated a preference for “real therapy” i.e. one-on-one sessions with a senior clinician (Table 3). While the intervention was unsuccessful, the study was well-described and executed and highlighted important factors (e.g. use of culturally salient interventions delivered by ethnic psychotherapist) that may facilitate successful interventions in future research in the target population of the study.

#### ***Other psychotherapeutic interventions***

Culturally adapted Behavioral Activation for Latinos (BAL) delivered in participant’s preferred language was evaluated in a pilot study that reported a decline in depressive symptomology over the study period of 20 weeks with large effect sizes on BDI-II (*d* = 1.07) and HRSD (*d* = 1.43). However, as reported by the study author, these results have to be interpreted with caution as the sample was very small, attrition rates were high and follow-up data was lacking [57].

Chu *et al.*[50] introduced a framework (Formative Method for Adapting Psychotherapies (FMAP)) for adapting evidence based interventions to diverse cultural groups. This framework was utilized to create Problem Solving Therapy—Chinese Older Adult (PSTCOA) that was piloted tested with a clinically depressed elderly Chinese woman who engaged in a 12-week program delivered in English. The intervention resulted in a decrease in depressive symptoms to sub-clinical levels and improvement in self-reported mood following 12 weeks of therapy; however no further follow up was reported. Although not described in detail, the qualitative data suggested that the intervention was acceptable to the client.

Schmaling *et al.*[60] likewise reported significant improvement in depression scores in Mexican Americans as a result of time-limited Problem Solving Therapy for Primary Care (PST-PC), delivered by bilingual therapists, in a dose-dependent manner; four or more PST-PC sessions resulted in greater reduction in Hopkins Symptom Checklist-20 (HSCL-20) scores relative to three or less PST-PC sessions (*t* (85) = -2.54, *p* = .05). Interestingly, while the treatment resulted in improvement in depression symptoms, it was not culturally adapted but rather provided as a generic intervention applicable to all cultural groups. Although a reasonable sample size was obtained, the lack of control group and attrition are limitations [60].

Beeber *et al.*[51] conducted a randomized controlled trial examining a short-term, in-home intervention using time limited, culturally tailored Inter-Personal Therapy (IPT) to reduce depressive symptoms in Latina mothers. The intervention involved over 11 in-home sessions with a psychiatric nurse and interpreter and five short booster sessions delivered by an interpreter. The intervention significantly reduced depressive symptoms compared to care as usual on the Center for Epidemiologic Studies Depression Scale (CES-D) measured across four time points. These sustained mean differences in CES-D scores were 4.1 points (*p* = .08) at T2, 8.3 points (*p* < .01) at T3, and 6.1 points (*p* = .04) at T4. However, the control group also experienced an improvement, which the authors attribute to the enhanced care of all participants. Qualitative results indicated participant satisfaction with the model, even when using an interpreter.

Cho *et al.*[52] employed the novel intervention logo-autobiography (LA) as a treatment modality for depression in Korean immigrant women. LA, based on Frankl’s existential psychology, incorporates autobiography as a therapeutic tool. It was found to be effective in reducing depressive symptoms in experimental groups relative to control groups immediately following the intervention and at the four week follow-up (*F* =6.832, *p* = .013; *F* =19.800, *p* ≤ .001); LA was in particular efficacious in non-medicated patients. It should be noted that patients were allowed to choose their own treatment conditions, which significantly affected the validity of results.

### Collaborative care models & exercise intervention

A small number of studies investigated the potential of using collaborative care models. Research in primary care settings has found a positive link between collaborative care and improvement in mental health care outcomes, including depression [75]. While most agree collaborative care is important and effective in patient management, the evidence is divided on the effectiveness of care management components for depression. This is highlighted in the study by Kwong *et al.*[33] that showed no advantage of adding a care management component relative to enhanced physician care in depressive symptomology.

Yeung *et al.*[64] investigated the feasibility of using a collaborative care model adapted to a cohort of depressed Chinese Americans using bilingual care managers in a randomized trial where the active phase ran over period of 24 weeks. There was a decrease in depressive symptoms in both control and intervention groups, but there was no significant difference between the groups. This could potentially be due to the fact that all participants were encouraged to speak to a mental health professional and many did engage in treatment regardless of group allocation, highlighting the potential of collaborative care models in engaging immigrants in mental health care.

In a smaller pilot randomized trial Uebelacker *et al.*[62] implemented a phone-based care management program with a stronger focus on depression assessment in the language of the participants and goal setting for Hispanic patients over a period of 12 weeks. Results only rendered a non-significant trend towards improvement in symptomology in the intervention group. Methodologically, the study was well designed but was limited by recruitment difficulties resulting in small sample size. In addition, there was limited participant engagement in the actual intervention with an average utilization rate of 1.7 phone calls out of 8 phone calls across the intervention group. The qualitative data suggested a mixed reception by participants, who on one hand felt the intervention was helpful, but on the other hand, thought it could have improved with face-to-face contact (Table 3).

In a unique study by Yeung *et al.*[63], the feasibility and efficacy of using Tai Chi to treat depressive symptomology in a Chinese-American cohort over a 12 week period was examined. The intervention was facilitated in Chinese and while results did show positive trend in remission rates, further research with larger sample is required to substantiate the results.

## Discussion

This review sought to identify the current literature on depression treatments and to evaluate the effectiveness of these treatments in first-generation immigrant populations. In recent years, the need for research of culturally salient interventions for mental health problems in ethnically diverse populations has been highlighted in the literature [76,77].

To date the majority of clinical research is still based on western, middle class, educated individuals and there is a dearth of studies on ethno-cultural groups [9]. This is reflected in the in the current review; while many high-quality studies were identified, the scope and breadth of available research is limited and under-representative of ethnic diversity and geographic locations.

Further, it is interesting to note that no pharmacological interventions were identified in our search considering that racial and ethnical differences may influence drug responses, which in turn relates to efficacy of medication prescribed for depression [10].

In our review a number of interventions were assessed including seven studies using CBT in a diverse range of treatment paradigms with mixed results. We conclude that culturally adapted psychotherapies and some non-culturally adapted therapies may have offered therapeutic benefits to immigrant populations assessed (primarily US-based Latino females). However, it was not possible to ascertain whether there was greater adherence to culturally adapted interventions relative to non-adapted interventions that were implemented in migrant populations; Renner *et al.*[59] reported that their non-culturally adapted CBT was not well-accepted in Turkish migrant women, whereas Schmaling *et al.*[60] implemented non-culturally adapted PST-PC in a Mexican sample with positive outcomes. So while there is growing interest in adaptation of interventions [12], it still remains to be determined whether or not culturally adapted interventions are more acceptable to migrants populations.

In the general population collaborative care models for depression have been shown to be successful in reducing depressive symptoms [78,79], yet the collaborative care studies [62,64] reviewed did not yield a significant effect on depressive symptoms relative to usual care. Similarly, while exercise has long been advocated as an effective treatment modality for depression [80] and a recent study recent study by Lavretsky *et al.*[81] showed the positive effects of Tai Chi on depression when complimenting anti-depressants, the Tai Chi study by Yeung *et al.*[63] only indicated a positive trend towards amelioration of depressive symptoms.

### Research implications

The generalizability of findings reported in the reviewed literature to other settings and ethnic groups is limited as sample sizes were generally small, some studies reported high attrition rates and the range of ethnic populations included in the studies was narrow. In addition, six of the 15 reviewed studies employed single group designs that can pose a threat to internal validity by overestimating the effectiveness of the intervention [82]. There was also insufficient reporting on processes for dealing with missing data in many studies and this further restricted the studies’ generalizability.

While the reviewed literature rendered mixed results about the efficacy of a number of psychotherapies, replication and extension of the current body of knowledge is needed including comparisons of the efficacy and acceptability of adapted with non-adapted interventions. For example, while there may be insufficient evidence that Tai Chi in its own right is an efficacious intervention for depression based on the results reported by Yeung *et al.*[63], prior research has provided support for the positive effects of physical exercise [80], including Tai Chi [81], on mental health including depression. Therefore further research of treatment paradigms that include Tai Chi as an adjunct treatment to medical or behavioral interventions, in migrant and non-migrant populations alike would extend the current knowledge base.

Additionally, while neither of the collaborative care studies reviewed could offer definitive results on the efficacy of their interventions, it may still be a worthwhile avenue for future research in migrant populations, especially given the success of collaborative care models on reducing depressive symptoms in the broader population; it is an avenue worth pursuing in future studies in migrant populations.

Regardless of the intervention of choice, future research should be cognizant of the research design and methods employed. In the current review only a small number of RCTs and pilot RCTs were identified across the different interventions, but further replication and extension of the current research by means of rigorous randomized clinical trials would significantly improve the current scientific evidence base. While this may not always be feasible for ethical or funding reasons, even replication of findings in larger, non-clinical trials can improve the cumulative body of knowledge. In addition, our review also accentuates the need for further research investigating depression treatment in first generation immigrants across ethnic groups and in diverse settings, not only limited to US-based studies.

### Limitations

Several limitations to the present review must be acknowledged. Importantly, the review only included peer-reviewed articles published from 2000 to early 2013 and while the utmost care was taken to perform a thorough search, the possibility that evidence might have been missed cannot be excluded. Further, as studies in which comorbidities were reported were excluded, it is possible that some evidence may have been missed through the selection process. In addition, as only English articles were included research published in other languages were excluded. Lastly, only studies investigating first-generation immigrant populations were included whereas the body of literature investigating ethnic minorities is much broader.

## Conclusion

To the best of our knowledge this is the first review to examine and evaluate the evidence on depression treatments in immigrant populations. As our review demonstrates, culturally-adapted CBT as well as other psychotherapies do hold considerable promise in reducing depressive symptoms in first-generation immigrant populations.

However, while our review accentuates the need for further research investigating depression treatment in first generation immigrants, we recognize that it is simplistic to merely call for further research to extend on the current body of knowledge. Researchers may want to improve conditions for migrant populations but face several barriers to undertaking this kind of work. As suggested by Minas [83] ethnic diversity and the mental health needs of immigrants that may differ from mainstream populations is still to permeate the consciousness of policy makers and major funding bodies. In addition, there is a tendency to relegate communities to “*other*” status, deviating from “whites” placing immigrants into “special needs” categories rather than viewing them as an integral part of the population who effectively are not having their mental health needs met [84,85]. Therefore, in order to broker greater collaboration between and within research institutions to validate existing knowledge and generate new evidence to address issues relating to immigrant mental health, research efforts need to be supported by policy and funding. Only then can we begin to develop effective evidence-based clinical and care interventions to meet the needs of population groups from increasingly diverse immigrant backgrounds.

This study did not require ethical approval.

## Competing interests

The authors declare that they have no competing interests.

## Authors’ contributions

BB and JA designed the review, collected and analyzed data and wrote the first draft of the paper. DM contributed to the design and subsequent stages of the write up. All authors read and approved the final manuscript.

## Pre-publication history

The pre-publication history for this paper can be accessed here:

http://www.biomedcentral.com/1471-244X/14/176/prepub

## References

[B1] WHO The Global Burden Of Disease: 2004 Update 2008 Switzerland: World Health Organization 27 49

[B2] Depression, Fact Sheet N°369 [http://www.who.int/mediacentre/factsheets/fs369/en/index.html]

[B3] Ahmed K Bhugra D Depression across ethnic minority cultures: diagnostic issues World Cult Psychiatry Res Rev 2007 2 47 56

[B4] Patel V Cultural factors and international epidemiology: depression and public health Br Med Bull 2001 57 1 33 45 11719922 10.1093/bmb/57.1.33

[B5] Berger JM Levant RF McMillan KK Kelleher W Sellers A Impact of gender role conflict, traditional masculinity ideology, alexithymia, and age on men’s attitudes toward psychological help seeking Psychol Men Masc 2008 6 1 73 78

[B6] Kirmayer LJ Cultural variations in the clinical presentation of depression and anxiety: implications for diagnosis and treatment J Clin Psychiatry 2001 62 Suppl 13 22 28 11434415

[B7] Kleinman A Culture and depression N Engl J Med 2004 351 10 951 953 15342799 10.1056/NEJMp048078

[B8] Bhugra D Migration and depression Acta Psychiatr Scand 2003 108 s418 67 72 10.1034/j.1600-0447.108.s418.14.x 12956818

[B9] Kirmayer L Narasiah L Munoz M Rashid M Ryder AG Guzder J Hassan G Rousseau C Pottie K Common mental health problems in immigrants and refugees: general approach in primary care Can Med Assoc J 2011 183 12 E959 E967 20603342 10.1503/cmaj.090292 PMC3168672

[B10] Schraufnagel TJ Wagner AW Miranda J Roy-Byrne PP Treating minority patients with depression and anxiety: what does the evidence tell us? Gen Hosp Psychiatry 2006 28 1 27 36 16377362 10.1016/j.genhosppsych.2005.07.002

[B11] Kalibatseva Z Leong FTL Depression among Asian Americans: review and recommendations Depress Res Treat 2011 2011 9 10.1155/2011/320902 PMC3180820 21961060

[B12] Bernal G Jiménez-Chafey MI Domenech Rodríguez MM Cultural adaptation of treatments: a resource for considering culture in evidence-based practice Prof Psychol Res Pr 2009 40 4 361

[B13] Thomas SL Thomas SD Displacement and health Br Med Bull 2004 69 115 127 15226201 10.1093/bmb/ldh009

[B14] Newbold B Health status and health care of immigrants in Canada: a longitudinal analysis J Health Serv Res Policy 2005 10 2 77 83 15831190 10.1258/1355819053559074

[B15] Dunn JR Dyck I Social determinants of health in Canada’s immigrant population: results from the national population health survey Soc Sci Med 2000 51 11 1573 1593 11072880 10.1016/s0277-9536(00)00053-8

[B16] Aguilera A Garza MJ Munoz RF Group cognitive-behavioral therapy for depression in spanish: culture-sensitive manualized treatment in practice J Clin Psychol 2010 66 8 857 867 20549680 10.1002/jclp.20706 PMC4167732

[B17] Baker-Ericzen MJ Connelly CD Hazen AL Duenas C Landsverk JA Horwitz SM A collaborative care telemedicine intervention to overcome treatment barriers for Latina women with depression during the perinatal period Fam Syst Health 2012 30 3 224 240 22709321 10.1037/a0028750 PMC3780578

[B18] Basoglu M Ekblad S Baarnhielm S Livanou M Cognitive-behavioral treatment of tortured asylum seekers: a case study J Anxiety Disord 2004 18 3 357 369 15125982 10.1016/S0887-6185(02)00248-7

[B19] Beeber LS Lewis VS Cooper C Maxwell L Sandelowski M Meeting the “now” need: PMH-APRN-Interpreter teams provide in-home mental health intervention for depressed Latina mothers with limited English proficiency J Am Psychiatry Nurses Assoc 2009 15 4 249 259 10.1177/1078390309344742 21665811

[B20] Carlsson JM Olsen DR Kastrup M Mortensen EL Late mental health changes in tortured refugees in multidisciplinary treatment J Nerv Ment Dis 2010 198 11 824 828 21048474 10.1097/NMD.0b013e3181f97be3

[B21] D’Angelo EJ Llerena-Quinn R Shapiro R Colon F Rodriguez P Gallagher K Beardslee WR Adaptation of the preventive intervention program for depression for use with predominantly low-income Latino families Fam Process 2009 48 2 269 291 19579909 10.1111/j.1545-5300.2009.01281.x

[B22] Drozek B Kamperman AM Bolwerk N Tol WA Kleber RJ Group therapy with male asylum seekers and refugees with posttraumatic stress disorder: a controlled comparison cohort study of three day-treatment programs J Nerv Ment Dis 2012 200 9 758 765 22922235 10.1097/NMD.0b013e318266f860

[B23] Duarte-Velez Y Bernal G Bonilla K Culturally adapted cognitive-behavior therapy: integrating sexual, spiritual, and family identities in an evidence-based treatment of a depressed Latino adolescent J Clin Psychol 2010 66 8 895 906 20568254 10.1002/jclp.20710

[B24] Fox PG Rossetti J Burns KR Popovich J Southeast Asian refugee children: a school-based mental health intervention Int J Psychiatr Nurs Res 2005 11 1 1227 1236 16268232

[B25] Gater R Waheed W Husain N Tomenson B Aseem S Creed F Social intervention for British Pakistani women with depression: randomised controlled trial Br J Psychiatry 2010 197 3 227 233 20807969 10.1192/bjp.bp.109.066845

[B26] Griffin K Connecting by telephone: depression care for rural latinos Northwest Publ Health 2012 29 1 22 23

[B27] Gross PK Nourse R Wasser TE Krulewicz S Effects of Paroxetine CR on depressive and anxiety symptoms in a community sample of adult hispanic women with major depression or generalized anxiety disorder Psychiatry 2006 3 5 64 68 21103179 PMC2990625

[B28] Heilemann MV Pieters HC Kehoe P Yang Q Schema therapy, motivational interviewing, and collaborative-mapping as treatment for depression among low income, second generation Latinas J Behav Ther Exp Psychiatry 2011 42 4 473 480 21619859 10.1016/j.jbtep.2011.05.001 PMC3152613

[B29] Interian A Ang A Gara MA Rodriguez MA Vega WA The long-term trajectory of depression among Latinos in primary care and its relationship to depression care disparities Gen Hosp Psychiatry 2011 33 2 94 101 21596201 10.1016/j.genhosppsych.2010.12.001 PMC3197230

[B30] Interian A Diaz-Martinez AM Considerations for culturally competent cognitive-behavioral therapy for depression with Hispanic patients Cogn Behav Pract 2007 14 1 84 97

[B31] Jones C Baker F Day T From healing rituals to music therapy: bridging the cultural divide between therapist and young Sudanese refugees Arts Psychother 2004 31 2 89 100

[B32] Kataoka SH Stein BD Jaycox LH Wong M Escudero P Tu W Zaragoza C Fink A A school-based mental health program for traumatized Latino immigrant children J Am Acad Child Adolesc Psychiatry 2003 42 3 311 318 12595784 10.1097/00004583-200303000-00011

[B33] Kwong K Chung H Cheal K Chou JC Chen T Depression care management for chinese americans in primary care: a feasibility pilot study Community Ment Health J 2011 49 2 157 165 22015960 10.1007/s10597-011-9459-9

[B34] La Roche MJ Batista C D’Angelo E A culturally competent relaxation intervention for latino/as: assessing a culturally specific match model Am J Orthopsychiatry 2011 81 4 535 542 21977939 10.1111/j.1939-0025.2011.01124.x

[B35] Le HN Zmuda J Perry DF Munoz RF Transforming an evidence-based intervention to prevent perinatal depression for low-income Latina immigrants Am J Orthopsychiatry 2010 80 1 34 45 20397987 10.1111/j.1939-0025.2010.01005.x

[B36] Le H-N Perry DF Stuart EA Randomized controlled trial of a preventive intervention for perinatal depression in high-risk Latinas J Consult Clin Psychol 2011 79 2 135 141 21319897 10.1037/a0022492

[B37] Miranda J Chung JY Green BL Krupnick J Siddique J Revicki DA Belin T Treating depression in predominantly low-income young minority women: a randomized controlled trial JAMA 2003 290 1 57 65 12837712 10.1001/jama.290.1.57

[B38] Mohlen H Parzer P Resch F Brunner R Psychosocial support for war-traumatized child and adolescent refugees: evaluation of a short-term treatment program Aust N Z J Psychiatry 2005 39 1–2 81 87 15660709 10.1080/j.1440-1614.2005.01513.x

[B39] Muñoz RF Le H-N Ippen CG Diaz MA Urizar GG Jr Soto J Mendelson T Delucchi K Lieberman AF Prevention of postpartum depression in low-income women: development of the mamás y bebés/mothers and babies course Cogn Behav Pract 2007 14 1 70 83

[B40] Nicolas G Arntz DL Hirsch B Schmiedigen A Correction to Nicolas *et al.* (2009) Prof Psychol Res Pr 2012 43 4 iv v

[B41] Paunovic N Ost LG Cognitive-behavior therapy vs exposure therapy in the treatment of PTSD in refugees Behav Res Ther 2001 39 10 1183 1197 11579988 10.1016/s0005-7967(00)00093-0

[B42] Saito T Kai I Takizawa A Effects of a program to prevent social isolation on loneliness, depression, and subjective well-being of older adults: a randomized trial among older migrants in Japan Arch Gerontol Geriatr 2012 55 3 539 547 22564362 10.1016/j.archger.2012.04.002

[B43] Santiago-Rivera AL Kanter JW Busch AM Rusch LC Reyes W West P Runge M Latino immigrants with depression: an initial examination of treatment issues at a community clinic J Immigr Minor Health 2011 13 4 772 779 20734139 10.1007/s10903-010-9380-2

[B44] Schwantes M McKinney C Music therapy with Mexican migrant farm workers: a pilot study Music Ther Perspect 2010 28 1 22 28

[B45] Shattell MM Quinlan-Colwell A Villalba J Ivers NN Mails M A cognitive-behavioral group therapy intervention with depressed Spanish-speaking Mexican women living in an emerging immigrant community in the United States Adv Nurs Sci 2010 33 2 158 169 10.1097/ANS.0b013e3181dbc63d 20460961

[B46] Smajkic A Weine S Djuric-Bijedic Z Boskailo E Lewis J Pavkovic I Sertraline, paroxetine, and venlafaxine in refugee posttraumatic stress disorder with depression symptoms J Trauma Stress 2001 14 3 445 452 11534876 10.1023/A:1011177420069

[B47] Somasundaram D Using cultural relaxation methods in post-trauma care among refugees in Australia Int J Cult Ment Health 2010 3 1 16 24

[B48] Markowitz JC Patel SR Balan IC Bell MA Blanco C Yellow Horse Brave Heart M Sosa SB Lewis-Fernandez R Toward an adaptation of interpersonal psychotherapy for Hispanic patients with DSM-IV major depressive disorder J Clin Psychiatry 2009 70 2 214 222 19192460 10.4088/jcp.08m04100 PMC8321621

[B49] Yeung A Hails K Chang T Trinh NH Fava M A study of the effectiveness of telepsychiatry-based culturally sensitive collaborative treatment of depressed Chinese Americans BMC Psychiatry 2011 11 154 21943315 10.1186/1471-244X-11-154 PMC3190334

[B50] Chu JP Huynh L Areán P Cultural adaptation of evidence-based practice utilizing an iterative stakeholder process and theoretical framework: problem solving therapy for Chinese older adults Int J Geriatr Psychiatry 2012 27 1 97 106 21500283 10.1002/gps.2698 PMC3239220

[B51] Beeber LS Holditch-Davis D Perreira K A Schwartz T Lewis V Blanchard H Canuso R Goldman BD Short-term in-home intervention reduces depressive symptoms in Early Head Start Latina mothers of infants and toddlers Res Nurs Health 2010 33 1 60 76 20043296 10.1002/nur.20363 PMC4096768

[B52] Cho S Bernstein KS Roh S Chen DC Logo-autobiography and its effectiveness on depressed korean immigrant women J Transcult Nurs 2013 24 1 33 42 22802301 10.1177/1043659612452005

[B53] Choi I Zou J Titov N Dear BF Li S Johnston L Andrews G Hunt C Culturally attuned Internet treatment for depression amongst Chinese Australians: a randomised controlled trial J Affect Disord 2012 136 3 459 468 22177742 10.1016/j.jad.2011.11.003

[B54] Dwight-Johnson M Aisenberg E Golinelli D Hong S O’Brien M Ludman E Telephone-based cognitive-behavioral therapy for latino patients living in rural areas: a randomized pilot study Psychiatr Serv 2011 62 8 936 942 21807834 10.1176/ps.62.8.pss6208_0936 PMC5515371

[B55] Gelman CR Lopez M Foster RP Evaluating the impact of a cognitive-behavioral intervention with depressed latinas: a preliminary report Soc Work Ment Health 2005 4 2 1 16

[B56] Interian A Allen LA Gara MA Escobar JI A pilot study of culturally adapted cognitive behavior therapy for Hispanics with major depression Cogn Behav Pract 2008 15 1 67 75

[B57] Kanter JW Santiago-Rivera AL Rusch LC Busch AM West P Initial outcomes of a culturally adapted behavioral activation for Latinas diagnosed with depression at a community clinic Behav Modif 2010 34 2 120 144 20176914 10.1177/0145445509359682

[B58] Piedra LM Byoun S-J Vida Alegre: preliminary findings of a depression intervention for immigrant Latino mothers Res Soc Work Pract 2012 22 2 138 150

[B59] Renner W Berry JW The ineffectiveness of group interventions for female Turkish migrants with recurrent depression Soc Behav Pers 2011 39 9 1217 1234 21976784 10.2224/sbp.2011.39.9.1217 PMC3184506

[B60] Schmaling KB Hernandez DV Problem-solving treatment for depression among Mexican Americans in primary care J Health Care Poor Underserved 2008 19 2 466 477 18469417 10.1353/hpu.0.0032

[B61] Tang P Gallagher-Thompson D Treatment of depressive symptoms in a Chinese female dementia caregiver: a case study using cognitive/behavioral methods Clin Gerontol 2005 28 3 81 85

[B62] Uebelacker LA Marootian BA Tigue P Haggarty R Primack JM Miller IW Telephone depression care management for Latino Medicaid health plan members: a pilot randomized controlled trial J Nerv Ment Dis 2011 199 9 678 683 21878782 10.1097/NMD.0b013e318229d100

[B63] Yeung A Lepoutre V Wayne P Yeh G Slipp LE Fava M Denninger JW Benson H Fricchione GL Tai Chi treatment for depression in Chinese Americans Am J Phys Med Rehabil 2012 91 10 863 870 22790795 10.1097/PHM.0b013e31825f1a67

[B64] Yeung A Shyu I Fisher L Wu S Yang H Fava M Culturally sensitive collaborative treatment for depressed chinese americans in primary care Am J Public Health 2010 100 12 2397 2402 20966373 10.2105/AJPH.2009.184911 PMC2978195

[B65] The Guidelines Manualhttp://www.nice.org.uk/guidelinesmanual

[B66] Crombie IK The Pocket Guide to Critical Appraisal: A Handbook for Health Care Professionals 1997 London: UK: BMJ

[B67] Critical Appraisal of A Case Study http://www.cebma.org/wp-content/uploads/Critical-Appraisal-Questions-for-a-Case-Study.pdf

[B68] Boaz A Ashby D Fit for Purpose? Assessing Research Quality For Evidence Based Policy And Practice 2003 London: ESRC UK Centre for Evidence Based Policy and Practice

[B69] McKnight PE McKnight KM Sidani S Figueredo AJ Missing Data: A Gentle Introduction 2007 New York: Guilford Press 18 40

[B70] Muñoz RF Mendelson T Toward evidence-based interventions for diverse populations: the San Francisco general hospital prevention and treatment manuals J Consult Clin Psych 2005 73 50 790 799 10.1037/0022-006X.73.5.790 16287379

[B71] Beck AT Dozois DJA Cognitive therapy: current status and future directions Annu Rev Med 2011 62 1 397 409 20690827 10.1146/annurev-med-052209-100032

[B72] Butler AC Chapman JE Forman EM Beck AT The empirical status of cognitive-behavioral therapy: a review of meta-analyses Clin Psychol Rev 2006 26 1 17 31 16199119 10.1016/j.cpr.2005.07.003

[B73] Lynch D Laws K McKenna P Cognitive behavioural therapy for major psychiatric disorder: does it really work? A meta-analytical review of well-controlled trials Psychol Med 2010 40 01 9 24 19476688 10.1017/S003329170900590X

[B74] Griffiths KM Farrer L Christensen H The efficacy of internet interventions for depression and anxiety disorders: a review of randomised controlled trials Med J Aust 2010 192 11 4 20528707 10.5694/j.1326-5377.2010.tb03685.x

[B75] Katon W Collaborative depression care models Am J Prev Med 2012 42 5 550 552 22516497 10.1016/j.amepre.2012.01.017

[B76] Alegria M Atkins M Farmer E Slaton E Stelk W One size does not fit all: taking diversity, culture and context seriously Adm Pol Ment Health 2010 37 1–2 48 60 10.1007/s10488-010-0283-2 PMC2874609 20165910

[B77] Department of Health and Human Services Mental Health: Culture, Race and Ethnicity-A supplement to the Mental Health: A report of the surgeon general 2001 Rockville, MD: Substance Abuse and Mental Health Services Administration (US) 159 168 20669516

[B78] Gilbody S Bower P Fletcher J Richards D Sutton AJ Collaborative care for depression: a cumulative meta-analysis and review of longer-term outcomes Arch Intern Med 2006 166 21 2314 17130383 10.1001/archinte.166.21.2314

[B79] Unützer J Katon W Callahan CM Williams JW Jr Hunkeler E Harpole L Hoffing M Della Penna RD Noël PH Lin EH Collaborative care management of late-life depression in the primary care setting JAMA 2002 288 22 2836 2845 12472325 10.1001/jama.288.22.2836

[B80] Mead GE Morley W Campbell P Greig CA McMurdo M Lawlor DA Exercise for depression Cochrane Database Syst Rev 2009 3 CD004366 19588354 10.1002/14651858.CD004366.pub4

[B81] Lavretsky H Altstein L Olmstead RE Ercoli L Riparetti-Brown M Cyr NS Irwin MR Complementary use of tai chi augments escitalopram treatment of geriatric depression: a randomized controlled trial Am J Geriatr Psychiatry 2011 19 10 839 21358389 10.1097/JGP.0b013e31820ee9ef PMC3136557

[B82] Griner D Smith TB Culturally adapted mental health interventions: a meta-analytic review Psychother Theor Res Pract Train 2007 43 4 531 548 10.1037/0033-3204.43.4.531 22122142

[B83] Minas H Kokanovic R Klimidis S Depression in multicultural Australia: policies, research and services Aust New Zealand Health Pol 2007 4 1 16 10.1186/1743-8462-4-16 PMC1964789 17645786

[B84] Delgado-Romero EA Galván N Maschino P Rowland M Race and ethnicity in empirical counseling and counseling psychology research: a 10-Year review Couns Psychol 2005 33 4 419 448

[B85] Ingleby D European Research on Migration and Health 2009 Brussels: International Organization for Migration (IOM)

